# The potential contribution of house crickets to the dietary zinc content and nutrient adequacy in young Kenyan children: a linear programming analysis using Optifood

**DOI:** 10.1017/S0007114522000915

**Published:** 2022-04-07

**Authors:** Hester L Coppoolse, Karin J Borgonjen-van den Berg, Prosper Chopera, Marijke Hummel, George Grimble, Inge Brouwer, Alida Melse-Boonstra

**Affiliations:** 1 Division of Human Nutrition and Health, Wageningen University & Research, Stippeneng 4, 6708 WE Wageningen, the Netherlands; 2 Division of Medicine, University College London, Gower Street, WC1E 6BT, London, UK; 3 Department of Nutrition Dietetics and Food Science, Faculty of Science, University of Zimbabwe, PO Box MP 169, Mt Pleasant, Harare, Zimbabwe

**Keywords:** Food-based dietary recommendations, Linear programming, Optifood, Kenyan children, Zinc deficiency, Nutrient adequacy, House crickets, Edible Insects

## Abstract

Zinc deficiency arising from inadequate dietary intake of bioavailable zinc is common in children in developing countries. Because house crickets are a rich source of zinc, their consumption could be an effective public health measure to combat zinc deficiency. This study used Optifood, a tool based on linear programming analysis, to develop food-based dietary recommendations (FBR) and predict whether dietary house crickets can improve both zinc and overall nutrient adequacy of children’s diets. Two quantitative, multi-pass 24-hour recalls from 47 children aged two and three years residing in rural Kenya were collected and used to derive model parameters, including a list of commonly consumed foods, median serving sizes, and frequency of consumption. Two scenarios were modelled: (i) FBR based on local available foods and (ii) FBR based on local available foods with house crickets. Results revealed that zinc would cease to be a problem nutrient when including house crickets to children’s diets (PRI coverage for zinc increased from 89% to 121% in the best-case scenario). FBR based on both scenarios could ensure nutrient adequacy for all nutrients except for fat, but energy percentage (E%) for fat was higher when house crickets were included in the diet (23 E% versus 19 E%). This manoeuvre, combined with realistic changes in dietary practices, could therefore improve dietary zinc content and ensure adequacy for twelve nutrients for Kenyan children. Further research is needed to render these theoretical recommendations, practical.

## Introduction

It is estimated that 17.3% of the world’s population is at risk of inadequate zinc intake, with the highest risk found in sub-Saharan African countries (1). Inadequate dietary intake of zinc and inefficient absorption from inhibitor-rich foods, such as cereal-based staples, are likely to be the primary causes of zinc deficiency, although illnesses that impair food intake, provoke catabolism or malabsorption, or increase zinc excretion are also associated with the development of zinc deficiency (2). Additional factors responsible for zinc deficiency in children less than five years of age include low body stores of zinc and increased zinc requirements for growth (3,4). During childhood, this deficiency is associated with impaired growth and development, suppressed immune function, and an increased risk of infections such as diarrhoea, malaria, and pneumonia. This may result in diminished cognitive performance, underweight, and stunting (3,5). Consequently, zinc deficiency is responsible for approximately 4% of under-five deaths, highlighting the importance of identifying ways to improve dietary zinc intake (6).

Dietary diversification with house crickets (*Acheta Domesticus*) may be an effective strategy to improve children’s dietary zinc intake (7). House crickets are nutrient-dense and a major source of zinc but consumption of house crickets is low in areas where diets are predominantly plant-based (8,9). Dietary diversification is a strategy that involves modifying food selection and food preparation patterns, in addition to increasing house cricket production in the area, to enhance the access to and utilization of house crickets (10). Although zinc bioavailability from house crickets is unknown and likely affected by, among others, the quantity of zinc, the protein content, and the presence of phytates in a meal, linear programming analysis can predict the potential contribution of house crickets to the zinc content of children’s diets while using a conservative estimate for zinc bioavailability levels (7,11). On the basis of dietary data collected from a target population, this mathematical modelling approach would allow identification of population-specific nutrient gaps, whilst also developing food-based dietary recommendations (FBR) that resemble current dietary practices but meet nutritional needs. It provides an objective method to predict the extent to which intervention strategies such as dietary diversification with house crickets can contribute to nutrient adequacy (12,13). No study to date has examined the effect on zinc content and overall nutrient adequacy of adding house crickets into FBR.

This study aims to determine the potential contribution of house crickets (*Acheta Domesticus*) to the dietary zinc content and nutrient adequacy in young children in Kisumu, western Kenya. In this area, young children are mostly fed on maize porridge, complemented with limited amounts of legumes and vegetables and only occasionally consume animal-based foods, including edible insects such as termites, grasshoppers, and crickets when in season.

## Methods

### Study design

The present study was based on cross-sectional dietary intake data collected as part of a randomized controlled trial on the effect of zinc fortified drinking water on zinc intake and bioavailability in children aged two to six years from rural western Kenya (14). Dietary intake data was collected to quantify young children’s zinc intake in- and outside their homes between February and August 2014. In a follow-up study, the same authors used linear programming to further optimize the zinc fortified drinking water scenario for children aged four to six years (12).

The original trial complied with the Declaration of Helsinki and approval was granted by the Ethical Review Committee of Kenyatta National Hospital/Nairobi University (KNH-ERC/A/335) and ETH Zurich Ethical Committee (EK 2013-N-31). Informed, signed consent was obtained from each household head and caregiver on behalf of their child.

### Setting

The study was conducted in Kisumu West District, western Kenya, in three villages in the sublocation Kajulu Koker. These three villages shared the same low socio-economic level and had a common unimproved water supply. The major livelihood in this area is subsistence farming or subsistence fishing in the parts bordering Lake Victoria (15,16). This area was selected because of the high incidence of child stunting (≥20%) and high prevalence of low plasma zinc concentrations (three out of four children had levels <650 μg/l or <570 μg/l collected in the morning and the afternoon, respectively), which was confirmed by pre-screening of a random sample of 303 children aged between 2 and 6 years old in the villages in September 2011 (17).

### Participants

The original trial involved 184 children aged two to six years residing in Kajulu Koker (14). Pre-intervention dietary intake data was collected from a subsample of 112 children. This sample size was estimated to be adequate to determine the mean daily zinc intake deviating less than 0.3 mg from the true intake with 80% power and 95% confidence, assuming an expected mean intake of 2.8 mg/day and a 10% nonresponse rate (14,18). For the present modelling study, children aged two and three years at baseline (n = 51) were chosen as the target group and their dietary intake data was used to develop FBR. This sample size is similar to previous studies using linear programming techniques and was found adequate to estimate population mean food serving sizes for commonly consumed foods (12).

Participants of the original trial were recruited at meetings in the village centres called by village chiefs where the study goals and design were presented. All residents of the villages could attend the meetings. Interested families were invited to a private interview where written consent was obtained. One child could participate per family and if more than one child in a household met the inclusion criteria, one was randomly selected using the random-number generation function in Microsoft Excel 2010 for Windows (Microsoft Corporation, USA). Children were excluded if they had severe anaemia (defined as a haemoglobin concentration <70.0 g/l, measured in the field using a photometer (HemoCue HB 201, Sweden)) or a chronic disease known to affect zinc metabolism, received zinc supplementation in the previous two weeks before the study, or medications known to interact with zinc metabolism (any type of regular medication for chronic diseases), or did not reside in the study area for the full study period. Children diagnosed with severe anaemia were referred for treatment with supplements.

### Data collection

#### Dietary intake

Data was collected by trained investigators who spoke the local language. Dietary intake was assessed using two quantitative, multi-pass 24-hour recalls per child, conducted on non-consecutive days. The multiple pass method includes the following steps: 1) obtain an unstructured, uninterrupted listing of all foods and beverages consumed; 2) help the respondent remembering foods with structured approaches to data collection, including memory cues (e.g. questions for specific types of foods, times and names of eating occasions, and reviews of foods for each eating occasion); and 3) ask a probing, unstructured question for any other foods recalled (19). Caregivers were asked to list the foods and drinks the child had consumed in the previous 24 hours and to include all ingredients and cooking methods for any mixed dishes. The quantity of foods, beverages, and ingredients consumed was assessed by weighing or, if not possible, by estimating with help of monetary value equivalents or household units in volume or size. For mixed dishes purchased or eaten outside the home, standard recipes were constructed and the quantity of single ingredients consumed by the child was calculated based on the proportions consumed. A detailed description of the dietary intake data collection has been published elsewhere (12).

The original trial also collected food price data based on participant-reports and interviews with food vendors from local markets but this data remained unpublished (14). Costs per 100 g edible portion of each food item were calculated.

#### Socio-demographic characteristics and anthropometry

Information on socio-economic and demographic characteristics of households were collected by using structured interviews. Weight and height were measured in duplicate to the nearest 0.1 kg and 0.1 cm, respectively, according to the World Health Organization (WHO) guidelines (20). Weight was measured using an electronic weighing scale (Ashton Meyers, England, United Kingdom), which was calibrated daily. Height was measured using a UNICEF wooden three-piece measuring board with a sliding head piece. Based on the WHO Child Growth Standards, children were classified as stunted and wasted if their height-for-age and weight-for-height-Z-score was less than minus 2 SD from the median value of the reference population, respectively (21).

### Blood sampling

Blood samples were collected at baseline for plasma zinc analysis according to the IZINCG protocol (22). The methods used are described in detail elsewhere (14). Plasma samples were sent to ETH Zurich, Switzerland, for analysis of plasma zinc concentrations using flame atomic absorption spectrometry. Proteins, including C-reactive protein, α1-acid glycoprotein, plasma ferritin, soluble transferrin receptor, and retinol binding protein, were analysed using a sandwich enzyme-linked immunosorbent assay technique in VitMin Laboratory, Germany (23). Haemoglobin was analysed in the field on a separate venous blood sample using a HemoCue photometer (HemoCure HB 201, Sweden). Zinc deficiency, vitamin A deficiency, anaemia, iron deficiency, and inflammation were defined as having a plasma zinc concentration ≤650 μg/l for blood samples collected during the morning (irrespective of fasting status) (24), a plasma retinol concentration <0.70 μmol/l (25), a haemoglobin concentration <110 g/l (26), a plasma ferritin concentration <12 μg/l and/or soluble transferrin receptor ≥10 mg/l (26,27), and a plasma concentration of C-reactive protein >5 mg/l and/or α1-acid glycoprotein >1.0 g/l (28), respectively. The plasma zinc, ferritin and retinol concentration values were not adjusted for sub-clinical inflammation.

### Data analysis

#### Habitual dietary intake

Energy and nutrient intakes were calculated from the 24-hour recalls using Compl-eat version 1.0 (Wageningen University, The Netherlands) and analysed using IBM SPSS version 24.0 (IBM, USA). Calculations were based on the food composition table (FCT) specifically developed for Kenyan children as described in detail by Kujinga et al. (12). Energy and the following thirteen nutrients were included: protein, fat, calcium, vitamin C, thiamine, riboflavin, niacin, vitamin B_6_, folate, vitamin B_12_, vitamin A, iron, and zinc. Normality of the distributions was tested visually using QQ plots. Outliers were identified for energy intake per participant according to the z-score method and participants were excluded from the analysis when their z-score for energy intake was above or below 2.58 because such values were considered implausible (29). Energy and nutrient intakes were adjusted for day-to-day variation according to the method developed by the National Research Council to calculate children’s habitual intakes (30,31). Habitual intakes were presented as median and range (25^th^, 75^th^ percentile of the distribution intakes). Prices per 100 g of edible food were used to estimate the average and 75^th^ percentile costs of the observed daily diet.

#### Preparation of model parameters

Data from 24-hour recalls of both days were used to generate model parameters in Microsoft Excel 2018 for Mac (Microsoft Corporation, USA), Microsoft Access 2019 (Microsoft Corporation, USA), and IBM SPSS version 24.0 (IBM, USA). Parameters included a list of non-condiment foods consumed by ≥5% of the target population, median serving size per food for those children who had consumed it, and minimum and maximum number of servings per week for each food (sub)group and single food.

Identical serving sizes for all similar food items within food subgroups were calculated as a weighted average of the median serving size of the raw edible portions based on 24-hour recalls and the number of children consuming the food. To illustrate, suppose that the food subgroup green leafy vegetables contains vegetable X, which is consumed by five children and has a median serving size of 50.0 g, and vegetable Y, which is consumed by ten children and has a median serving size of 25.0 g, the serving size for both vegetables in the food subgroup would be 33.3 g. This method was applied to avoid bias in the selection of specific food items towards the selection of larger or smaller serving sizes reported for those specific food items (32). The minimum and maximum number of servings per week for each food, food subgroup, and food group were based on the 5^th^ and 95^th^ percentile distributions of serving counts, respectively. The average number of servings per food group was defined as the 50^th^ percentile. Foods with a maximum number of servings per week below 1 were excluded.

House crickets (*Acheta Domesticus*) were added in portions of 46.5 g based on a serving containing a fixed number of 100 crickets of assumed average weight, with a maximum number of servings per week set at seven, assuming they would not be consumed more than once a day (33). Based on participant-reported data and interviews with food vendors from markets in the area, the price of house crickets was estimated to be 25 Kenyan Shilling (KES)/100 g edible portion (1 KES = 0.01 US$ in July 2020). Zinc content for house crickets was set at 6.71 mg/100 g (33). Details of the nutrient composition of house crickets per 100 g edible portion can be found in Supplementary Appendix Table 1.

Less frequently consumed food items were added if they were nutrient-dense. Nutrient-dense foods were defined as (i) foods that were consumed by less than 5% of the target population and contributed ≥30% to the intake of a nutrient with inadequate levels per median serving size; and (ii) foods consumed by more than 5% of the target population that contributed ≥20% to the intake of a nutrient with inadequate levels and for which an increase in the number of servings per week was assumed to be feasible (27).

The FCT developed for Kenyan children was also used in Optifood. An energy constraint was introduced to ensure all modelled diets provided the average energy requirement for the target group. Energy requirements were estimated using the participant’s mean body weight and the European Food Safety Authority (EFSA) algorithm (34). The participant’s mean body weight was compared to the EFSA reference weights of population groups in Europe to ensure it was representative of the population (35). Energy requirements for adjusted physical activity levels (PAL) for growth (PAL = 1.4) were used (34). In addition, thirteen nutrients were identified as potential problem nutrients for Kenyan children and selected for analysis for nutrient adequacy, including: protein, fat, calcium, vitamin C, thiamine, riboflavin, niacin, vitamin B_6_, folate, vitamin B_12_, vitamin A, iron, and zinc (12). The EFSA Population Reference Intakes (PRIs) were used for all thirteen nutrients, except for fat and vitamin B12 (36–46). For fat, a reference intake of 35 energy% (E%) was used (47). EFSA Adequate Intake levels were used for vitamin B_12_ because the EFSA PRI is not available for this nutrient (48). Given that animal-source food consumption was meagre in the target group, the EFSA PRIs for zinc and iron were adapted to reflect a low bioavailability level of 15% and 5%, respectively (49). These conservative bioavailability levels were also chosen for house crickets. Population-weighted energy and nutrient requirements were calculated to reflect the requirements for the different ages and sexes that made up the target population. Habitual energy and nutrient intakes were compared with these theoretical requirements and coverage for each nutrient was determined.

#### Linear programming analysis using Optifood

Optifood linear programming software version 4.0.9.0 was used to identify problem nutrients and develop population-specific FBR (50). Two dietary scenarios were modelled: (i) the daily diet comprising all foods consumed without house crickets and (ii) the daily diet complemented with house crickets. Optifood Module 1 to 3 were run for both scenarios. Module 1 was run to check the feasibility of the diets for the target population. In this module, twenty-one different seven-day diets were generated based on the model parameters and reviewed to determine if they were realistic with energy contents within a sufficient range to allow for modelling (51). If any of the generated diets was considered unrealistic, one or more of the constraint levels (including the energy content; the minimum and maximum number of servings from food (sub)groups per week; and the minimum and maximum g of single food items) were changed (52).

Module 2 was run to develop the two best seven-day diets: one diet optimized close to the average food pattern and one diet optimized close to the thirteen nutrient PRIs constrained by the minimum and maximum number of servings per week, the Module 2 ‘nutritionally best diet’. The total number of nutrients achieving 100% of the PRI in the Module 2 nutritionally best diet were counted per scenario and compared between the scenarios to determine whether including house crickets could potentially improve nutritional adequacy of the diet. Results from the Module 2 nutritionally best diet analysis were used to identify individual FBR of food (sub)groups and individual foods contributing at least 5% to the nutrient intake for any of the nutrients considered to formulate FBR for testing in the Module 3 analysis.

Module 3 was run to test alternative sets of FBR to select the best combination of recommendations for the target group. In Module 3, twenty-six modelled seven-day diets were generated of which thirteen diets had a maximised content of one of the thirteen nutrients (selecting the high-nutrient-dense foods within each food group to verify the highest possible nutrient intake, the ‘best-case scenario’) and thirteen diets had a minimised content of one of the thirteen nutrients (selecting the low-nutrient-dense foods per food group to verify the lowest possible nutrient intake, the ‘worst-case scenario’). Module 3 was run in three phases. In phase I, a “no recommendation diet” was run to identify problem nutrients, which were defined as nutrients reaching <100% of the PRI in the best-case scenario without recommendations. Phase II was run to achieve nutrient adequacy for those nutrients that were unable to reach 70% in the phase I worst-case scenario. Nutrient adequacy was defined as a nutrient level reaching ≥70% of the PRI in the worst-case scenario, since nutrient intakes achieving ≥70% of the PRI predict a low risk of inadequate intakes in the population (53). In phase III, FBR incorporating selected nutrient-dense foods were tested.

Specifically, in phase II, individual FBR identified in the Module 2 nutritionally best diet were added separately to the model and compared to identify FBR that, when combined, likely provided the highest number of nutrients reaching PRIs ≥70% in the worst-case scenario for the seven-day diet. Individual FBR incorporating food groups were preferred to individual FBR incorporating food subgroups and food subgroups were preferred to foods as consumers generally find it easier to follow guidance on food groups than to strictly implement guidance on specific foods (54). All possible combinations of the selected FBR were tested and the combination that contained the fewest number of FBR and generated the highest number of nutrients reaching ≥70% of the PRI for the lowest cost was selected.

In the last phase of Module 3, nutrient-dense foods as identified in the Module 2 nutritionally best diet were incorporated into the FBR and tested separately to determine if they improved nutrient adequacy for those nutrients that were unable to reach PRIs ≥70% in the phase II worst-case scenario for the seven-day diet. Nutrient-dense foods were added individually at various frequencies to the final FBR. Finally, the set of recommendations that achieved ≥70% of the PRI in the worst-case scenario for most nutrients and that was below the mean of current daily diet cost was selected.

## Results

### Participant characteristics

The model parameters were based on data for 47 out of 51 children aged two and three years in Kisumu West District. Reasons for excluding data were that the child had completed only one dietary recall (n = 3) and that the child had implausible daily energy intakes (n = 1). Children were on average 37 months old and their mean body weight was 13.4 kg (Table 1). In total, 61.7% of the children were girls and the percentage prevalence of stunting was 19.1%.

Over half of the children (55.3%) were zinc deficient and inflammation affected 70.2% of the children. Missing data from micronutrient markers other than zinc and haemoglobin occurred in three children because of failure to draw sufficient blood for all biomarker analyses in these children.

### Food and zinc intake

Data analysis included ninety-four 24-hour recalls, including first and second recalls. In total, 74 non-condiment foods were reported in the dietary recalls over two days. Excluded condiments were soup powder, lemon powder, and baking soda. From this list, 28 foods were excluded because they were consumed by <5% of the children and these included manufactured foods (e.g. chocolate drinking powder). Next, 12 foods were excluded for having a maximum consumption frequency per week below 1. Overall, 34 foods were included in the modelling (shown in Table 2). Foods consumed by over 75% of children included cooking oil (93.6%), white maize flour (89.4%), fried tomato (89.4%), and fried stem onion (87.2%). Fruit consumption was absent, except for mango. Serving sizes for similar food items within food subgroups varied from 4.22 g/day for the food subgroup other vegetables to 81.9 g/day for the food subgroup whole grains and products. All vegetables were consumed in portion sizes below 30 g/day, except for cabbage that had a median serving size of 88.0 g/day. Details of the median serving sizes and costs for individual foods are described in Supplementary Appendix Table 2.

Habitual zinc intake was 4.49 mg per child per day, covering 52.2% of the PRI for zinc. An overview of the energy and nutrient requirements used to define problem nutrients and nutrient adequacy is shown in Supplementary Appendix Table 3. Habitual intakes and PRI coverage for energy and the remaining twelve nutrients can be found in Supplementary Appendix Table 4. The average daily diet cost was 52 KES, the 75^th^ percentile of cost was 62 KES, and costs ranged from 22 KES to 110 KES (1 KES = 0.01 US$ in July 2020).

### Linear programming

In Module 1, twenty-one realistic diets were generated for each scenario and no changes in constraint levels were needed. The Module 2 optimized diet close to the average food pattern covered 74.5% of the PRI for zinc, which improved to 78.2% when the diet was complemented with house crickets (shown in Table 3). The Module 2 nutritionally best diet covered 100% of the PRI for seven of the thirteen nutrients in the scenario without house crickets and for ten of the thirteen nutrients in the scenario with house crickets. Daily costs ranged from 47 KES to 56 KES in the diets without house crickets and from 48 KES to 69 KES in the diets with house crickets.

In the Module 3 best-case scenario without house crickets and without recommendations (phase I), fat, vitamin B12, vitamin A, and zinc were identified as problem nutrients (shown in Table 4). In phase II, FBR identified in the Module 2 ‘nutritional best diet’ were added and these included: added fats (9 servings/week), dairy products (7 servings/week), fruits (5 servings/week), cowpea leaves (3 servings/week), legumes (2 servings/week), omena dagaa (4 servings/week), provitamin A rich dark green leafy vegetables (3 servings/week), and whole grains and products (24 servings/week), evenly divided over finger millet flour (7 servings/week) and yellow maize flour (7 servings/week). These recommendations covered 70.8% of the PRI for zinc in the worst-case scenario and ensured nutrient adequacy for all nutrients except for fat and vitamin A, which remained at <20 E% and <60% of the PRI, respectively.

Nutrient-dense foods that contributed ≥20% to the intake of problem nutrients were oil for fat and mango for vitamin A. Foods consumed by less than 5% of the children that contributed ≥30% to the intake of problem nutrients per serving size (calculated as a weighted average of the median serving size of similar food items within the food subgroup and the number of children consuming the food) were sour cow’s milk (294.5 g) and butternut (176 g) for vitamin A. Addition of four servings of mango per week improved PRI coverage for vitamin A to 72.8%, whereas addition of oil, sour cow’s milk, or butternut did not increase the number of nutrients achieving ≥70% of the PRI. Instead, inclusion of more than four weekly servings of oil, two weekly servings of butternut or one weekly serving of sour cow’s milk in the FBR would exceed the energy constraint. The combination of additional oil and additional mango did not increase the number of nutrients achieving ≥70% of the PRI, but would increase the daily cost of the diet. The final set of FBR hypothetically ensured nutrient adequacy at the population level in the worst-case scenario for all nutrients except for fat, which remained at 18.7 E%. In the best-case scenario, PRI coverage remained <100% of the PRI for niacin, vitamin B_12_, vitamin A, iron, and zinc and <25 E% for fat. Daily costs for this scenario with FBR including nutrient-dense foods ranged from 51 KES in the worst-case scenario to 58 KES in the best-case scenario (shown in Table 4).

In the scenario with house crickets, problem nutrients were fat and vitamin A (shown in Table 5). Based on the Module 2 nutritionally best diet, a set of FBR was developed for this scenario and included: added fats (11 servings/week), dairy products (7 servings/week), fruits (5 servings/week), house crickets (3 servings/week), cowpea leaves (3 servings/week), provitamin A rich dark green leafy vegetables (3 servings/week), whole grains and products (24 servings/week) of which finger millet flour (7 servings/week) and yellow maize flour (7 servings/week). These recommendations covered 76.5% of the PRI for zinc in the worst-case scenario and ensured nutrient adequacy for all nutrients except for fat and vitamin A, which remained at <25 E% and <70% of the PRI in the worst-case scenario, respectively. Nutrient-dense foods that contributed ≥20% to the intake of problem nutrients were oil for fat and mango for vitamin A. Sour cow’s milk and butternut were consumed by less than 5% of the children and contributed ≥30% to the intake of vitamin A per median serving size. Addition of six servings of oil per week improved PRI coverage for fat to 24.8 E% and addition of four servings of mango per week improved PRI coverage for vitamin A to 73.1% of the PRI. On the other hand, addition of sour cow’s milk or butternut did not increase the number of nutrients achieving ≥70% of the PRI and inclusion of more than one weekly serving of sour cow’s milk or butternut in the FBR would exceed the energy constraint. Also, the combination of additional oil and additional mango would exceed the energy requirement. The final set of FBR hypothetically ensured nutrient adequacy at the population level for all nutrients except for fat, which remained at 22.6 E%. Daily costs for this scenario with FBR including nutrient-dense foods ranged from 53 KES in the worst-case scenario to 60 KES in the best-case scenario (shown in Table 5).

**Figure 1:**
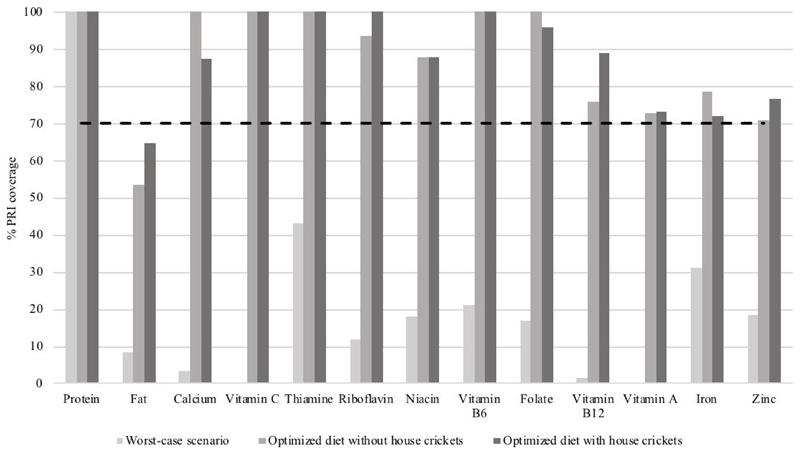
Comparison of the percentage Population Reference Intake (PRI) coverage* for the two scenarios† (FBR based on local available foods and FBR with house crickets) against the worst-case scenario using linear programming (Optifood) among young children (n = 47) aged two and three years in Kisumu, western Kenya. Values above the dashed line (70%) indicate nutrient adequacy. * values capped at 100%. † final set of FBR in worst-case scenario. FBR, food-based dietary recommendations.

The final set of FBR for both scenarios in comparison to the best diet within the average food pattern are shown in Table 6. In both scenarios, the number of servings selected of grains and grain products and fruits slightly exceeded the number observed in the average food pattern, whereas the number of servings of vegetables selected was below the number observed in the average food pattern. Three weekly servings of house crickets were selected in the optimal modelled diet with house crickets and this recommendation replaced two weekly servings of legumes and four weekly servings of fish. Comparison of the percentage PRI coverage for the final set of FBR for both scenarios (FBR based on local available foods and FBR with house crickets) against the worst-case scenario is shown in Figure 1. FBR based on both scenarios could ensure nutrient adequacy for all nutrients except for fat, but E% of fat was highest in the scenario with house crickets (22.6 E% versus 18.7 E%).

## Discussion

This linear programming analysis explored the potential contribution of house crickets to the dietary zinc content and nutrient adequacy in young Kenyan children. Data presented show that zinc would cease to be a problem nutrient when including house crickets to children’s diets (PRI coverage for zinc increased from 89.4% to 121% in the best-case scenario) in this population where zinc deficiency is common. Further analysis indicated that three weekly servings of house crickets provided in portions of 46.5 g could replace recommendations for four weekly servings of small, whole fish with bones (e.g. omena dagaa (*Rastrineobola argentea*)) and two weekly servings of legumes. Although FBR in both scenarios could ensure nutrient adequacy for all thirteen nutrients except for fat, FBR with house crickets were superior to FBR without house crickets at reaching E% of fat (22.6 E% versus 18.7 E% in the worst-case scenario) at 2 KES additional cost (0.02 US$^1^). These results provide an evidence base that justifies further testing of interventions with house crickets to meet dietary (zinc) requirements within the existing dietary pattern of young Kenyan children.

These FBR are based on children’s actual dietary patterns, and the foods recommended are assumed to be available, affordable, and acceptable for the target population (55). However, the complexity and specificity of this linear programming analysis may have resulted in FBR that are not in line with dietary recommendations, such as our recommendation of only six servings of vegetables per week. The analysis was based on meeting the requirements for thirteen selected nutrients rather than for all of the essential nutrients (51). Although these nutrients were considered to be potential problem nutrients for children in this area, results may have been different when other nutrients, such as dietary fibre, were included (12,56). In addition to the assumptions made about nutrient requirements, the final set of FBR developed in this study was based on the few vegetables that were reported to be consumed in only limited amounts (e.g. both provitamin A rich dark leafy green vegetables and vitamin C-rich vegetables had serving sizes of approximately 25 g). More nutrient-dense foods from food groups other than vegetables were preferred. Likewise, FBR with house crickets would replace four weekly servings of fish and two weekly servings of legumes, a recommendation that may not fit within the local agri-food culture. Thereby, these FBR suggest a trade-off in dietary diversity to improve nutrient adequacy, a finding that is contradictory to the current state of knowledge (57). Results of this study should therefore not be viewed as dietary recommendations to guide the public, instead, they should be interpreted as a theoretical indication of the potential contribution of house crickets to reach nutrient adequacy. They pave the way for more extensive research, such as clinical trials, on the effectiveness and acceptability of consumption of dietary house crickets to prevent zinc deficiency in young Kenyan children.

Crickets, ants, termites, and grasshoppers are traditionally consumed around Lake Victoria, either as an occasional delicacy or as a replacement of food in times of shortages (58,59). Insects are well accepted, but their actual consumption varies between communities (60–62). Although processing insects is not yet common in Kenya, an experimental study on the acceptability of biscuits containing 10% crickets among primary school children in the Nyanza District in western Kenya showed that cricket biscuits had good sensory acceptability, which could be explained by the high number of children (76%) who consumed insects when they were in season (63). Similarly, an experimental study on the acceptability of cereal-cricket porridge in children aged 3-5 years old showed that porridge flour containing maize, millet, and cricket power was liked very much and that young children can develop a greater liking for it with continued exposure over time (64). Most of the insects consumed in the area are harvested from wild habitats and, as such, are restricted to certain localities and subject to seasonal availability (58,65). Commercial insect production can ease the constraints of seasonal cricket availability and lower their sale price, which is currently relatively high (11.6 KES per portion of 46.5 g house crickets as compared to 4.6 KES per portion of 14.2 g omena dagaa) (66). It should be noted, however, that insects are susceptible to microbiological hazards if proper heat treatment or storage conditions are not applied and that there are currently no specific standards for the use of insects as food in Kenya (65,67). Therefore, food safety issues associated with the process of scaling up the insect sector should be identified and managed to deliver to the consumer edible insects that are safe (8). The practicality of this is discussed more fully, elsewhere (68).

The current analysis revealed that among two- and three-year-old children in Kisumu, western Kenya, the intake of fat, vitamin B12, vitamin A, and zinc is below the requirements. These problem nutrients correspond to the high levels of vitamin A and zinc deficiency, being 36.2% and 55.3%, respectively. Even with a conservative estimate for iron and zinc bioavailability from crickets, only fat and vitamin A remained problem nutrients when house crickets were included in the diet (49,69). Vitamin A fortification of cooking oil is mandatory in Kenya but fortified products were not yet consumed by the study participants. Promotion of the use and consumption of vitamin A fortified oil could be effective to eliminate the deficit in fat and vitamin A intake. Remaining nutrient gaps could mostly be covered by adapting the local diet to include nutrient-dense foods, however, incorporation of such FBR results in 2.9-fold increase in the cost of the diet if all foods are purchased. Although this increase is still below the 75^th^ percentile cost of the observed daily diet, this increase may reduce the affordability of such diets to the high proportion of the households in the area that live in poverty. Therefore, interventions that focus on lowering the cost of house crickets, such as rearing or farming crickets, should be explored (66).

This study has several limitations. First, the validity of the conclusions drawn from the Optifood analysis depends on the accuracy of the model parameters, including dietary intake data (51). In this study, dietary intake data was collected based on recall from the caregiver, a method that can introduce recall bias. Second, the cross-sectional data captured a snapshot of the dietary intake during the preharvest season (12). For example, mango is a seasonal product that was included in the final set of FBR and its availability and consumption patterns will differ in the postharvest season. Therefore, results cannot be extrapolated to other agricultural seasons and comparative analyses using dietary intake data from different seasons are required to understand how FBR would change throughout the year (12). Third, identical serving sizes for all similar food items within food subgroups were created to avoid bias in selection of food items based on serving size. However, this method does not take into account that these differences in serving size may be caused by the food item’s affordability and/or acceptability to children or their parents. Fourth, the presence of concurrent inflammation can lead to overestimation of the true prevalence of zinc deficiency in the study population (70). Finally, the sample size was small, meaning that errors in portion size estimates may have occurred (71). Although the study had insufficient power to allow generalization to the larger Kenyan population, it nevertheless captured the foods most commonly consumed in the locality.

In summary, a set of seven FBR with house crickets could ensure nutrient adequacy for twelve of the thirteen nutrients modelled, including zinc. These results can serve as a guide for designing culturally acceptable, population-specific interventions with house crickets to meet the zinc and overall nutrient requirements of young children in western Kenya. Various initiatives to stimulate commercial insect production and insect consumption have already been implemented in the area. However, several obstacles to the widespread use of insects as human food to combat nutritional deficiencies remain. First, information on the bioavailability level of iron and zinc from edible insects is required to revise and update current FBR. In addition, issues regarding the microbial safety of edible insects should be considered. Finally, if insects are to become part of the habitual diets, large quantities of insects will need to be available on a continuous basis.

## Supplementary Material

Supplementary Material

## Figures and Tables

**Table 1:** Nutritional status of children (n = 47) aged two and three years in Kisumu, western Kenya*.

Background characteristics	Value
Age, months	37.1 ± 6.71
Sex (female), *n* (%)	29 (61.7)
Weight, kg	13.4 ± 2.5
Height-for-age, z-score	-1.02 ± 1.48
Stunted†, *n* (%)	9 (19.1)
Weight-for-height, z-score	0.02 ± 0.89
Wasted†, *n* (%)	1 (2.1)
Plasma zinc concentration, μg/l	652±131
Zinc deficiency‡, *n* (%)	26 (55.3)
Plasma retinol concentration, μmol/l§	0.82 ± 0.28
Vitamin A deficiency∥, *n* (%)§	17 (36.2)
Haemoglobin concentration g/l	104 ± 12.0
Anaemia¶, *n* (%)	30 (63.8)
Iron deficiency**, *n* (%)§	19 (40.4)
Inflammation††, *n* (%)§	33 (70.2)

n, number; SD, standard deviation.

* values are presented as mean and standard deviation unless stated otherwise.

† based on the World Health Organization Child Growth Standards (21).

‡ plasma zinc concentration <650 μg/l (24).

§ n = 44 due to missing data from three children.

∥ plasma retinol concentration <0.70 μmol/l (25).

¶ haemoglobin concentration <110 g/l (26).

** plasma ferritin concentration <12 μg/l and/or soluble transferrin receptor ≥10 mg/l (26,27).

†† plasma concentration of C-reactive protein >5 mg/l and/or α1-acid glycoprotein >1.0 g/l (28).

**Table 2:** Foods consumed by children (n = 47) aged two and three years*, percentage of children who consumed the food, median serving sizes, average and maximum servings per week, and costs in Kisumu, western Kenya.

Food group, subgroup, and food	% of children consuming	Median serving size (g/d)†	Average / Max servings per week‡
Added fats			7 / 11
Butter, ghee, margarine, unfortified			7
Cooking fat	42.6	7.75	7
Vegetable oil, unfortified			7
Cooking oil	93.6	17.3	7
Added sugars			0 / 7
Sugar, unfortified			7
White sugar	27.7	36.0	7
Bakery and breakfast cereals			0 / 2
Refined grain bread, unenriched/unfortified			2
White bread	12.8	66.0	1
Dairy products			6 / 8
Fluid or powdered milk, unfortified			7
Whole cow’s milk, fresh, boiled	74.5	71.0	7
Fruits			0 / 7
Vitamin C rich fruit			7
Mango, ripe, peeled	25.5	44.5	5
Grains and grain products			14 / 32
Enriched/fortified grains and products, whole or refined			7
Tropicana wheat flour, baked	51.1	11.0	7
Refined grains and products, unenriched/unfortified			2
White rice, boiled	17.0	42.5	2
Whole grains and products, unenriched/unfortified			27
Finger millet flour, boiled	55.3	81.9	7
Maize, fresh, roasted	36.2	81.9	7
Sorghum flour, boiled	29.8	81.9	7
White maize flour, boiled	89.4	81.9	7
White maize grains, dried, boiled	46.8	81.9	3
White wheat flour, baked	53.2	81.9	7
Whole white maize flour, boiled	74.5	81.9	7
Yellow maize flour, boiled	29.8	81.9	7
Yellow maize grains, dried, boiled	12.8	81.9	1
Legumes, nuts, and seeds			0 / 3
Cooked beans, lentils, peas			3
Cocoa rose beans, dried, boiled	19.2	51.1	1
White beans, dried, boiled	14.9	51.1	1
Meat, fish, and eggs			1 / 4
Eggs			1
Egg, baked	10.6	36.0	1
Small, whole fish with bones			4
Fulu fish, fried	10.6	14.2	1
Omena dagaa, dried, boiled	21.3	14.2	3
Omena dagaa, dried, fried	29.8	14.2	3
Fish without bones			4
Nile perch, boiled	12.8	18.5	2
Nile perch, fried	8.51	18.5	1
Vegetables			15 / 24
Other vegetables			14
Red bulb onion, fried	19.2	4.22	7
Stem onion, boiled	44.7	4.22	7
Stem onion, fried	87.2	4.22	7
Vitamin A source dark green leafy vegetables			14
Sukuma wiki, fried	48.9	24.0	3 3
Vitamin C-rich vegetables			14
Cabbage, fried	25.5	25.2	2
Cowpea leaves, boiled	36.2	25.2	3
Okra, boiled	25.5	25.2	3
Tomato, boiled	27.7	25.2	7
Tomato, fried	89.4	25.2	7

* all foods consumed by at least 5% of the children.

† serving sizes of similar food items within food subgroups are calculated as a weighted average of the median serving sizes of the raw edible portions based on 24-hour recalls and the number of children consuming the food.

‡ values in the 95th percentile of distribution.

**Table 3:** Nutrient composition as percentage of the population reference intake (PRI) of the two scenarios for the average* and the nutritionally best diet† (Module 2) modelled using linear programming (Optifood) among young children (n = 47) aged two and three years in Kisumu, western Kenya.

	No house crickets		House crickets	
	Average food pattern (% of the PRI)	Nutritionally best diet (% of the PRI)	Average food pattern (% of the PRI)‡	Nutritionally best diet (% of the PRI)§
Protein	212	242	216	301
Fat (E%)	22.2	25.2	22.3	27.4
Calcium	97.9	141	85.9	131
Vitamin C	100	179	100	186
Thiamine	198	217	198	172
Riboflavin	88.3	100	125	359
Niacin	69.9	90.6	71.2	100
Vitamin B6	132	135	133	135
Folate	70.0	124	69.6	104
Vitamin B12	30.4	76.0	38.8	221
Vitamin A	29.4	57.8	29.7	57.5
Iron	84.6	81.1	84.2	95.0
Zinc	74.5	74.9	78.2	104
Cost per day in KES	46.90	56.40	47.90	69.40
Number of nutrients ≥100% of the PRI	4	7	5	10

KES, Kenyan Shilling.

* best diet within average food pattern closest to median food pattern of the population.

† best diet deviating from average food pattern constrained by the minimum and maximum servings per week.

‡ 1 serving of house crickets per week.

§ 7 servings of house crickets per week.

**Table 4:** Results for the scenario without house crickets and without or with food-based dietary recommendations (FBR) modelled using linear programming (Optifood) among young children (n = 47) aged two and three years in Kisumu, western Kenya.

	Protein (% of the PRI)	Fat (E%)	Calcium (% of the PRI)	Vitamin C (% of the PRI)	Thiamine (% of the PRI)	Riboflavin (% of the PRI)	Niacin (% of the PRI)	Vitamin B6 (% of the PRI)	Folate (% of the PRI)	Vitamin B12 (% of the PRI)	Vitamin A (% of the PRI)	Iron (% of the PRI)	Zinc (% of the PRI)	Cost in KES per day
No house cricket diet without FBR														
Best-case scenario	311	29.1	156	192	271	110	109	161	142	76.3	62.4	113	89.4	64.9
Worst-case scenario	145	2.91	3.60	0.10	43.0	12.1	18.2	21.2	17.0	1.70	0.00	31.1	18.5	21.0
No house cricket diet with FBR*														
Worst-case scenario	250	18.7	137	136	186	91.9	84.2	120	108	75.8	52.4	78.2	70.8	48.3
Worst-case scenario results for nutrient-dense foods added to the no house cricket diet with FBR														
FBR + oil 4 servings/week	250	20.9	138	139	190	92.5	86.3	120	111	75.8	52.5	78.2	70.8	49.2
FBR + mango 4 servings/week†	251	18.7	139	177	192	93.6	87.9	123	119	76.0	72.8	78.6	71.0	51.1
FBR + sour cow’s milk 1 servings/week	258	19.6	150	140	190	99.3	85.9	120	112	84.9	58.2	78.5	73.2	50.2
FBR + butternut 2 servings/week	259	18.7	140	164	197	94.5	100	129	120	76.1	63.4	79.7	72.6	52.9
FBR + oil 3 servings/week + fruits 9 servings/week	256	19.8	141	179	194	94.3	95.4	124	126	76.0	72.8	78.6	71.0	52.2
Best-case scenario results for the final set of FBR selected in the no house cricket diet	261	20.2	146	229	230	102	97.7	140	138	76.3	87.2	90.7	78.6	58.1

PRI, population reference intake; KES, Kenyan Shilling.

* FBR: added fats (9 servings/week); dairy products (7 servings/week); fruits (5 servings/week); cowpea leaves (3 servings/week); legumes (2 servings/week); omena dagaa (4 servings/week); vitamin A source dark green leafy vegetables (3 servings/week); whole grains and products (24 servings/week) of which finger millet flour (7 servings/week) and yellow maize flour (7 servings/week).

† final set of FBR selected

**Table 5:** Results for the scenario with house crickets and without or with food-based dietary recommendations (FBR) modelled using linear programming (Optifood) among young children (n = 47) aged two and three years in Kisumu, western Kenya.

	Protein (% of the PRI)	Fat (E%)	Calcium (% of the PRI)	Vitamin C (% of the PRI)	Thiamine (% of the PRI)	Riboflavin (% of the PRI)	Niacin (% of the PRI)	Vitamin B6 (% of the PRI)	Folate (% of the PRI)	Vitamin B12 (% of the PRI)	Vitamin A (% of the PRI)	Iron (% of the PRI)	Zinc (% of the PRI)	Cost in KES per day
House cricket diet without FBR													
Best-case scenario	375	31.0	160	199	271	371	133	171	143	243	64.0	116	121*	74.8
Worst-case scenario	145	2.91	3.60	0.10	41.6	12.1	18.2	21.2	14.7	1.70	0.00	31.1	18.5†	21.0
House cricket diet with FBR‡													
Worst-case scenario	228	22.6	86.0	139	177	198	84.4	117	85.9	88.8	52.8	71.8	76.5	50.5
Worst-case scenario results for nutrient-dense foods added to the house cricket diet with FBR													
FBR + oil 6 servings/week	230	24.8	87.1	142	181	199	89.4	118	90.5	88.9	52.8	71.8	76.5	51.7
FBR + mango 4 servings/week§	229	22.6	87.3	180	183	199	88.0	120	96.0	89.0	73.1	72.2	76.7	53.3
FBR + sour cow’s milk 1 servings/week	236	23.5	98.2	143	181	205	86.1	118	89.8	97.9	58.5	72.1	78.9	52.5
FBR + butternut 1 servings/week	231	22.6	86.7	153	182	199	90.4	121	90.6	89.0	58.2	72.6	77.4	52.5
Best-case scenario results for the final set of FBR selected in the house cricket diet	252	23.9	109	227	218	240	99.4	136	114	114	84.7	83.7	86.0	60.0

PRI, population reference intake; KES, Kenyan Shilling.

* 0 servings of house crickets per week.

† 7 servings of house crickets per week.

‡ FBR: added fats (11 servings/week); dairy products (7 servings/week); fruits (5 servings/week); house crickets (3 servings/week); cowpea leaves (3 servings/week); vitamin A source dark green leafy vegetables (3 servings/week); whole grains and products (24 servings/week) of which finger millet flour (7 servings/week) and yellow maize flour (7 servings/week).

§ final set of FBR selected.

**Table 6:** Food-based dietary recommendations (FBR) developed in the scenario without house crickets and with house crickets in comparison with the best diet within the average food pattern among young children (n = 47) aged two and three years in Kisumu, western Kenya.

	Average food pattern (servings/week)*	Final set of FBR selected in the scenario without house crickets† (servings/week)	Final set of FBR selected in the scenario with house crickets‡ (servings/week)
Added fats	11	9	11
Dairy products	7	7	7
Grains and grain products	23	24	24
Fruits	5	9	9
Legumes, nuts and seeds	2	2	NA
Meat, fish, and eggs	4	4	3
Vegetables	24	6	6

NA, not applicable.

* results of the Module 2 best diet within average food pattern of the scenario without house crickets.

† results of the Module 3 analysis of the scenario without house crickets and with FBR including nutrient-dense foods.

‡ results of the Module 3 analysis of the scenario with house crickets (3 servings/week in portions of 46.5 g) and with FBR including nutrient-dense foods.

## Footnotes

1 1 KES = 0.01 US$ in July 2020
