# Supplementary material for: The potential contribution of house crickets to the dietary zinc content and nutrient adequacy in young Kenyan children: a linear programming analysis using Optifood
Source: Br J Nutr. Author manuscript; Available in PMC 2023 Feb 14. (PMC9876809; doi:10.1017/S0007114522000915)
Supplement: Supplementary Material [file EMS143904-supplement-Supplementary_Material.pdf]

3

Supplementary Material

Supplementary Appendix Table 1. Nutrient composition of house crickets* per 100 g edible portion.

Nutrient	Median
Energy (kJ)	585
Protein (g)	20.5
Fat (g)	6.8
Calcium (mg)	40.7
Vitamin C (mg)	3.0
Thiamine (mg)	0.04
Riboflavin (mg)	3.41
Niacin (mg)	3.84
Vitamin B6 (mg)	0.23
Folate (μg DFE)	1.50
Vitamin B12 (μg)	5.37
Vitamin A (μg RAE)	9
Iron (mg)	1.93
Zinc (mg)	6.71

* on an as is basis (33).

Supplementary Appendix Table 2. Foods consumed* by children (n = 47) aged two to three years, median serving sizes and costs in Kisumu, western Kenya.

Food group, subgroup, and food	Median serving size (g/day)†	Cost (KES/100 g)‡
Added fats		
Butter, ghee, margarine, unfortified		
Cooking fat	7.75	20.46
Vegetable oil, unfortified		
Cooking oil	17.3	24.33
Added sugars		
Sugar, unfortified		
White sugar	36.0	11.01
Bakery and breakfast cereals		
Refined grain bread, unenriched/unfortified		
White bread	66.0	10.77
Dairy products		
Fluid or powdered milk, unfortified		
Whole cow's milk, fresh, boiled	71.0	6.25
Fruits		
Vitamin C rich fruit		
Mango, ripe, peeled	44.5	11.19
Grains and grain products		
Enriched/fortified grains and products, whole or refined		
Tropicana wheat flour, raw, baked	11.0	7.38
Refined grains and products, unenriched/unfortified		
White rice, raw, boiled	42.5	12.74
Whole grains and products, unenriched/unfortified		
Finger millet flour, raw, boiled	33.0	23.07
Maize, fresh, roasted	105	5.28
Sorghum flour, raw, boiled	39.0	9.96
White maize flour, raw, boiled	108	7.26
White maize grains, dried, boiled	88.5	5.28
White wheat flour, raw, baked	10.0	7.38
Whole white maize flour, raw, boiled	116	7.26
Yellow maize flour, raw, boiled	153	8.12
Yellow maize grains, dried boiled	58.0	6.25
Legumes, nuts, and seeds		
Cooked beans, lentils, peas		
Cocoa rose beans, dried, boiled	31.0	9.20
White beans, dried, boiled	77.0	9.09
Meat, fish, and eggs		
Eggs		
Egg, raw, baked	36.0	37.08
Small, whole fish with bones		

Fulu fish, raw, fried	22.0	39.13
Omena Dagaa, dried, boiled	13.3	32.22
Omena Dagaa, dried, fried	12.0	32.22
Fish without bones		
Nile perch, raw, boiled	16.5	53.22
Nile perch, raw, fried	21.5	53.22
Vegetables		
Other vegetables		
Red bulb onion, raw, fried	8.00	15.41
Stem onion, raw, boiled	4.00	10.76
Stem onion, raw, fried	3.50	10.76
Vitamin A source dark green leafy vegetables		
Sukuma wiki, raw, fried	24.0	11.29
Vitamin C-rich vegetables		
Cabbage, raw, fried	88.0	4.44
Cowpea leaves, raw, boiled	30.0	5.80
Okra, raw, boiled	3.50	32.10
Tomato raw, boiled	6.00	10.50
Tomato, raw, fried	17.5	10.50

* all foods consumed by at least 5% of the children.
† values are median serving sizes of the raw edible portions based on 24-hour recalls.
‡ mean costs in Kenyan Shilling (KES) per 100g of edible portion estimated from market survey data and participant-reported data.

Supplementary Appendix Table 3. Population reference intakes (PRIs)* for the target population consisting of girls aged two years (n = 13), girls aged three years (n = 16), boys aged two years (n = 8), and boys aged three years (n = 10).

Nutrient	Requirement
Energy (kJ)	4682†
Fat‡ (E%)	35.0
Protein (g)	12.5†
Calcium (mg)	450
Vitamin C (mg)	20.0
Thiamine (mg)	0.43§
Riboflavin (mg)	0.60
Niacin (mg)	6.86§
Vitamin B6 (mg)	0.60
Folate (μg DFE)	120
Vitamin B12‖ (μg)	1.50
Vitamin A (μg RAE)	250
Iron (mg)	14.0¶
Zinc (mg)	8.60**

E%, percentage of energy intake; DFE, dietary folate equivalents; RAE, retinol activity equivalents.
* values are based on the European Food Safety Authority (EFSA) PRIs and adapted to reflect the requirements for the different ages and sexes that make up the target population unless stated otherwise.
† corresponds to a body weight of 13.4 kg.
‡ based on the EFSA reference intake range for fat (47).
§ corresponds to an energy requirement of 4682 kJ.
‖ based on the EFSA adequate intake for vitamin B12 (48).
¶ corresponds to the World Health Organization (WHO) low bioavailability level of 5% (49).
** corresponds to the WHO low bioavailability level of 15% (49).

Supplementary Appendix Table 4. Habitual daily energy and nutrient intakes* adjusted for day-to-day variation† and population reference intake (PRI) coverage without house crickets for young children (n = 47) aged two and three years in Kisumu, western Kenya‡

Nutrient	Median	Range	% of PRI
Energy (kJ)	5527	4339, 7364	118%
Protein (g)	33.7	25.0, 44.8	270%
Calcium (mg)	376	198, 681	83.6%
Vitamin C (mg)	21.5	9.00, 39.0	108%
Thiamine (mg)	0.70	0.49, 1.12	162%
Riboflavin (mg)	0.45	0.27, 0.67	74.2%
Niacin (mg)	4.85	2.90, 7.05	70.7%
Vitamin B6 (mg)	0.59	0.35, 0.97	99.0%
Folate (μg DFE)	92.1	54.9, 152	76.8%
Vitamin B12 (μg)	0.57	0.16, 1.51	38.0%
Vitamin A (μg RAE)	69.5	31.5, 131	27.8%

Iron (mg)	10.1	7.85, 13.8	72.1%
Zinc (mg)	4.49	3.07, 7.32	52.2%

* based on 24-hour recalls.
† according to methods developed by the National Research Council (30,31).
‡ values are presented as median and range (25th, 75th percentile of the distribution intakes).
