# Supplementary material for: The potential contribution of house crickets to the dietary zinc content and nutrient adequacy in young Kenyan children: a linear programming analysis using Optifood
Source: Br J Nutr. 2022 Apr 7;129(3):478–90. doi: 10.1017/S0007114522000915 (PMC9876809; doi:10.1017/S0007114522000915)
Supplement: Supplementary file 1 [file S0007114522000915sup001.docx]

# Supplementary Material

| **Supplementary Appendix Table 1.** Nutrient composition of house crickets^*^ per 100 g edible portion. |  |
| --- | --- |
| Nutrient | Median |
| Energy (kJ) | 585 |
| Protein (g) | 20.5 |
| Fat (g) | 6.8 |
| Calcium (mg) | 40.7 |
| Vitamin C (mg) | 3.0 |
| Thiamine (mg) | 0.04 |
| Riboflavin (mg) | 3.41 |
| Niacin (mg) | 3.84 |
| Vitamin B6 (mg) | 0.23 |
| Folate (μg DFE) | 1.50 |
| Vitamin B12 (μg) | 5.37 |
| Vitamin A (μg RAE) | 9 |
| Iron (mg) | 1.93 |
| Zinc (mg) | 6.71 |
| * on an as is basis (33). |  |

	

| Supplementary Appendix Table 2. Foods consumed^*^ by children (n = 47) aged two to three years, median serving sizes and costs in Kisumu, western Kenya. |  |  |
| --- | --- | --- |
| Food group, subgroup, and food | Median serving size (g/day)^†^ | Cost (KES/100 g)^‡^ |
| Added fats |  |  |
| Butter, ghee, margarine, unfortified |  |  |
| Cooking fat | 7.75 | 20.46 |
| Vegetable oil, unfortified |  |  |
| Cooking oil | 17.3 | 24.33 |
| Added sugars |  |  |
| Sugar, unfortified |  |  |
| White sugar | 36.0 | 11.01 |
| Bakery and breakfast cereals |  |  |
| Refined grain bread, unenriched/unfortified |  |  |
| White bread | 66.0 | 10.77 |
| Dairy products |  |  |
| Fluid or powdered milk, unfortified |  |  |
| Whole cow’s milk, fresh, boiled | 71.0 | 6.25 |
| Fruits |  |  |
| Vitamin C rich fruit |  |  |
| Mango, ripe, peeled | 44.5 | 11.19 |
| Grains and grain products |  |  |
| Enriched/fortified grains and products, whole or refined |  |  |
| Tropicana wheat flour, raw, baked | 11.0 | 7.38 |
| Refined grains and products, unenriched/unfortified |  |  |
| White rice, raw, boiled | 42.5 | 12.74 |
| Whole grains and products, unenriched/unfortified |  |  |
| Finger millet flour, raw, boiled | 33.0 | 23.07 |
| Maize, fresh, roasted | 105 | 5.28 |
| Sorghum flour, raw, boiled | 39.0 | 9.96 |
| White maize flour, raw, boiled | 108 | 7.26 |
| White maize grains, dried, boiled | 88.5 | 5.28 |
| White wheat flour, raw, baked | 10.0 | 7.38 |
| Whole white maize flour, raw, boiled | 116 | 7.26 |
| Yellow maize flour, raw, boiled | 153 | 8.12 |
| Yellow maize grains, dried boiled | 58.0 | 6.25 |
| Legumes, nuts, and seeds |  |  |
| Cooked beans, lentils, peas |  |  |
| Cocoa rose beans, dried, boiled | 31.0 | 9.20 |
| White beans, dried, boiled | 77.0 | 9.09 |
| Meat, fish, and eggs |  |  |
| Eggs |  |  |
| Egg, raw, baked | 36.0 | 37.08 |
| Small, whole fish with bones |  |  |
| Fulu fish, raw, fried | 22.0 | 39.13 |
| Omena Dagaa, dried, boiled | 13.3 | 32.22 |
| Omena Dagaa, dried, fried | 12.0 | 32.22 |
| Fish without bones |  |  |
| Nile perch, raw, boiled | 16.5 | 53.22 |
| Nile perch, raw, fried | 21.5 | 53.22 |
| Vegetables |  |  |
| Other vegetables |  |  |
| Red bulb onion, raw, fried | 8.00 | 15.41 |
| Stem onion, raw, boiled | 4.00 | 10.76 |
| Stem onion, raw, fried | 3.50 | 10.76 |
| Vitamin A source dark green leafy vegetables |  |  |
| Sukuma wiki, raw, fried | 24.0 | 11.29 |
| Vitamin C-rich vegetables |  |  |
| Cabbage, raw, fried | 88.0 | 4.44 |
| Cowpea leaves, raw, boiled | 30.0 | 5.80 |
| Okra, raw, boiled | 3.50 | 32.10 |
| Tomato raw, boiled | 6.00 | 10.50 |
| Tomato, raw, fried | 17.5 | 10.50 |
| * all foods consumed by at least 5% of the children. † values are median serving sizes of the raw edible portions based on 24-hour recalls. ‡ mean costs in Kenyan Shilling (KES) per 100g of edible portion estimated from market survey data and participant-reported data. |  |  |

| **Supplementary Appendix Table 3.** Population reference intakes (PRIs)* for the target population consisting of girls aged two years (n = 13), girls aged three years (n = 16), boys aged two years (n = 8), and boys aged three years (n = 10). |  |
| --- | --- |
| Nutrient | Requirement |
| Energy (kJ) | 4682† |
| Fat‡ (E%) | 35.0 |
| Protein (g) | 12.5† |
| Calcium (mg) | 450 |
| Vitamin C (mg) | 20.0 |
| Thiamine (mg) | 0.43§ |
| Riboflavin (mg) | 0.60 |
| Niacin (mg) | 6.86§ |
| Vitamin B6 (mg) | 0.60 |
| Folate (μg DFE) | 120 |
| Vitamin B12││ (μg) | 1.50 |
| Vitamin A (μg RAE) | 250 |
| Iron (mg) | 14.0¶ |
| Zinc (mg) | 8.60** |
| E%, percentage of energy intake; DFE, dietary folate equivalents; RAE, retinol activity equivalents. * values are based on the European Food Safety Authority (EFSA) PRIs and adapted to reflect the requirements for the different ages and sexes that make up the target population unless stated otherwise. † corresponds to a body weight of 13.4 kg. ‡ based on the EFSA reference intake range for fat (47). § corresponds to an energy requirement of 4682 kJ. ││ based on the EFSA adequate intake for vitamin B12 (48). ¶ corresponds to the World Health Organization (WHO) low bioavailability level of 5% (49). ** corresponds to the WHO low bioavailability level of 15% (49). |  |

| **Supplementary Appendix Table 4.** Habitual daily energy and nutrient intakes^*^ adjusted for day-to-day variation^† ^and population reference intake (PRI) coverage without house crickets for young children (n = 47) aged two and three years in Kisumu, western Kenya^‡^ |  |  |  |
| --- | --- | --- | --- |
| Nutrient | Median | Range | % of PRI |
| Energy (kJ) | 5527 | 4339, 7364 | 118% |
| Protein (g) | 33.7 | 25.0, 44.8 | 270% |
| Calcium (mg) | 376 | 198, 681 | 83.6% |
| Vitamin C (mg) | 21.5 | 9.00, 39.0 | 108% |
| Thiamine (mg) | 0.70 | 0.49, 1.12 | 162% |
| Riboflavin (mg) | 0.45 | 0.27, 0.67 | 74.2% |
| Niacin (mg) | 4.85 | 2.90, 7.05 | 70.7% |
| Vitamin B6 (mg) | 0.59 | 0.35, 0.97 | 99.0% |
| Folate (μg DFE) | 92.1 | 54.9, 152 | 76.8% |
| Vitamin B12 (μg) | 0.57 | 0.16, 1.51 | 38.0% |
| Vitamin A (μg RAE) | 69.5 | 31.5, 131 | 27.8% |
| Iron (mg) | 10.1 | 7.85, 13.8 | 72.1% |
| Zinc (mg) | 4.49 | 3.07, 7.32 | 52.2% |
| * based on 24-hour recalls. † according to methods developed by the National Research Council (30,31). ‡ values are presented as median and range (25th, 75th percentile of the distribution intakes). |  |  |  |
